# Intramuscular deposit of calcium is a potential reason for hypocalcaemia in COVID-19

**DOI:** 10.1007/s12020-021-02708-y

**Published:** 2021-04-08

**Authors:** Johannes Kalbhenn, Julian Knoerlein

**Affiliations:** Department of Anaesthesiology and Critical Care, Medical Center—University of Freiburg, Faculty of Medicine, University of Freiburg, Freiburg, Germany

To the Editor:

With great interest we read the letter of Singh et al. specifying several aetiologies for hypocalcaemia in coronavirus disease 19 (COVID-19) patients [1]. Hypocalcaemia is common in COVID-19 [1] and associated with decreased survical [2]. In the following by reporting the case of a patient treated with severe COVID-19 at our department we propose an additional mechanism leading to hypocalcaemia: a 44-year-old male patient with severe respiratory failure due to COVID-19 was referred to the intensive care unit for extracorporeal membrane oxygenation. On admission he presented with hypocalcaemia probably caused by the abovementioned mechanisms. During the first four weeks of therapy he needed excessive substitution of calcium-gluconate to keep serum-calcium level within the normal range (Fig. 1). Overall, more than 5.400 ml of calcium-gluconate 10% solution had to be infused. Computed tomography of the abdomen two weeks after referral coincidentally showed cloudy radiopaque deposits in the midst of the gluteal muscles (Fig. 2B) which were most likely caused by calcium. Compared to the computed tomography on admission (Fig. 2A) this was a new finding. We believe that precipitation of calcium in the muscle was aggravated by the excessive substitution of calcium-gluconate solution during therapy.

**Fig. 1:**
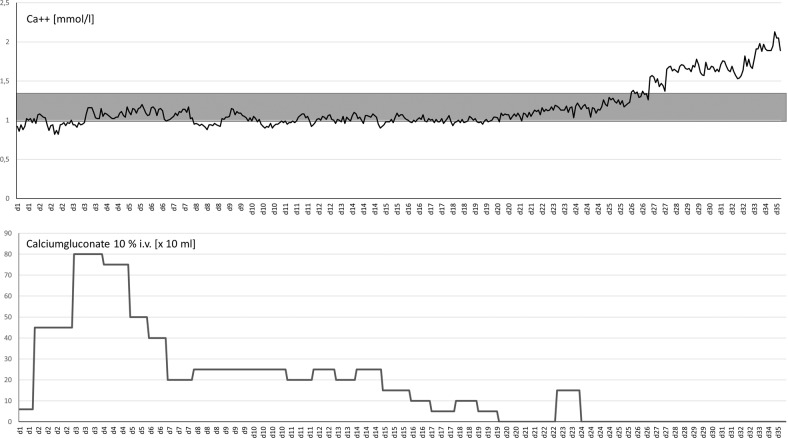
Upper graph: Serum-concentration of calcium from day 1 to day 35 of treatment (grey area indicating normal range). Lower graph: Amount of substituted calcium-gluconate 10% solution per day

**Fig. 2:**
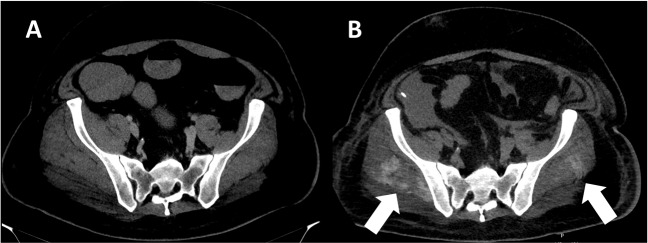
Computed tomography on admission (panel **A**) and 2 weeks later (panel **B**). White arrows indicating radiopaque depots of precipitated calcium within gluteal muscles

We found calcium precipitates also in the deltoid muscle but not in other organs. COVID-19 often is accompanied by myositis [3]. It is obvious that negatively charged valences, for example, from myoglobin are presented when muscle tissue loses its integrity and that calcium ions may accumulate and calcium salts may precipitate especially in these regions. Precipitation of calcium should be considered an additional possible reason for hypocalcaemia in COVID-19.

## Data Availability

On request.
